# Initial Experience With Transoral Endoscopic Thyroidectomy via the Submental and Vestibular Approach for the Treatment of Thyroid Cancer: A Retrospective Cohort Study

**DOI:** 10.3389/fsurg.2022.882150

**Published:** 2022-07-20

**Authors:** Yuanyuan Wang, Yilong Fu, Guoyang Wu, Yezhe Luo, Chaolong Yan, Jinbo Fu, Suqiong Lin

**Affiliations:** ^1^ Department of Thyroid Surgery, Zhengzhou University First Affiliated Hospital, Zhengzhou, China; ^2^ Department of General Surgery, Zhongshan Hospital, Xiamen University, Xiamen, China

**Keywords:** thyroid, thyroid cancer, thyroidectomy, transoral endoscopic thyroidectomy, submental approach

## Abstract

**Background:**

Transoral endoscopic thyroidectomy vestibular approach is feasible and safe but has some unavoidable limitations, such as sensory changes in the center of the chin region. We aim to report our initial experience in performing transoral endoscopic thyroidectomy via the submental and vestibular approach for the treatment of thyroid cancer.

**Patients and Methods:**

This retrospective cohort study included patients with thyroid cancer confirmed by fine-needle aspiration who underwent endoscopic thyroidectomy and central lymph node dissection via the submental and vestibular approaches between November 2019 and January 2020. Patients’ clinicopathological characteristics, operation details, and postoperative complications were analyzed.

**Results:**

Fifteen surgeries were performed successfully. The mean ± standard deviation age of the patients was 37 ± 10.8 years, the average duration of surgery was 146.5 ± 34.6 min, and the median intraoperative blood loss was 11.1 ± 6.3 mL. None of the surgeries were converted to open thyroidectomy. According to postoperative pathology, all cases involved papillary thyroid carcinoma or papillary thyroid microcarcinoma. One patient developed transient recurrent laryngeal nerve paralysis. No patient developed skin numbness at the center of the chin region.

**Conclusions:**

Transoral endoscopic thyroidectomy via the submental and vestibular approach is effective and safe in patients with thyroid cancer and does not lead to skin numbness at the center of the chin region. This technique is beneficial for surgeons less experienced in performing transoral thyroid surgery as it involves using a short and direct route to the thyroid gland, which can reduce the difficulty in establishing the first operative space to some extent.

## Introduction

The ever-increasing incidence of thyroid cancer is becoming one of the major public health issues worldwide, possibly due to improved clinical examination and changes in environmental exposures. The global age-standardized incidence rate of thyroid cancer increased by 20% between 1990 and 2013 (1). Scar tissue resulting from open thyroidectomy performed to treat thyroid cancer can cause serious mental distress to patients, especially young women. Hence, endoscopic thyroidectomy has played an important role in treating thyroid disease over the last 20 years. The first transoral endoscopic thyroid surgery was performed by Wilhelm et al. (2) in 2009. Fu et al. completed the first transoral endoscopic thyroidectomy in China in November 2011 (3). Transoral endoscopic thyroidectomy vestibular approach (TOETVA) is feasible and safe (4–6), but has some unavoidable limitations such as sensory changes in the center of the chin region and limited ability to extract large tumors.

Recently, Chen et al. (7) reported performing a transoral and submental thyroidectomy for a patient with a cytologically benign thyroid nodule to avoid injury to the mental nerve. Perigli et al. (8) also reported their experience on twenty-two patients underwent this hybrid access. Our team started performing transoral endoscopic thyroidectomy via the submental and vestibular approach (TOETSMVA) in November 2019. In this study, we aim to report our initial experience with TOETSMVA for the treatment of thyroid cancer.

## Materials and Methods

We retrospectively reviewed the records of patients who underwent TOETSMVA and central lymph node dissection by the same surgeon between November 2019 and January 2020. All patients were diagnosed with papillary thyroid cancer (PTC) based on fine-needle aspiration results. All preoperative examinations indicated the absence of lateral lymph node metastases and distant metastases. The clinicopathological characteristics, operation details, and postoperative complications of the patients were analyzed. The potential for patient selection bias was mitigated by enrolling all patients who had undergone TOETSMVA during the predefined study period.

According to the 2015 American Thyroid Association guidelines for adult patients with differentiated thyroid cancer (9), other related guidelines, and our experience with endoscopic surgery, we set the following eligibility criteria for TOETSMVA: (1) a differentiated thyroid tumor smaller than 2 cm in diameter without lymph node metastases or distant metastases; (2) no surgical and radiological history of head and neck cancer; (3) no hyperthyroidism or hypothyroidism; (4) no history of abnormal physical scarring. Patients were excluded from receiving TOETSMVA if they had the below conditions: (1) preference for the complete absence of scarring in the anterior cervical region; (2) oral or cervical skin infections; and (3) preoperative examinations indicative of the tumor having invaded adjacent structures such as the trachea, esophagus, and recurrent laryngeal nerve (RLN).

The study received approval from the Medical Ethics Committee of our institution. The need for informed consent was waived owing to the retrospective nature of the study.

### Preoperative Preparation

**Figure 1:**
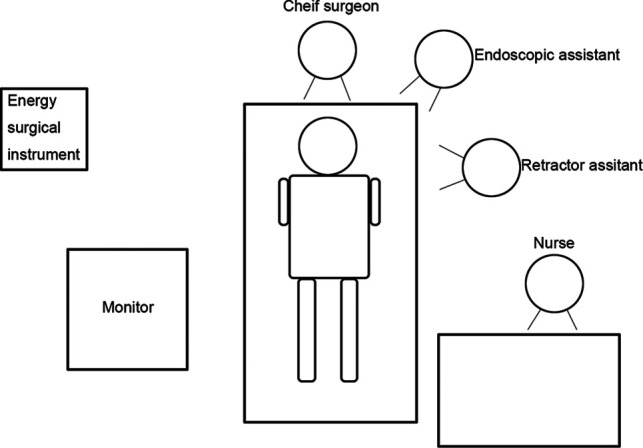
The positions of Surgeons, nurse and endoscopic monitor.

A prophylactic course of antibiotic (Cefazolin Sodium, 2 g with 0.9% normal saline, 100 mL) was initiated 30 min before the operation (10, 11). After satisfactory induction of general anesthesia, the patient was placed in the supine position with the neck slightly extended. Subsequently, an endotracheal tube embedded with neuromonitoring electrodes was used for intubation via the oral route by placement on the right side of the neck. After disinfection of the surgical area and draping, all the instruments were connected and checked. The positions of surgeons, nurse and endoscopic monitor were in the Figure 1.

### Step One

**Figure 2:**
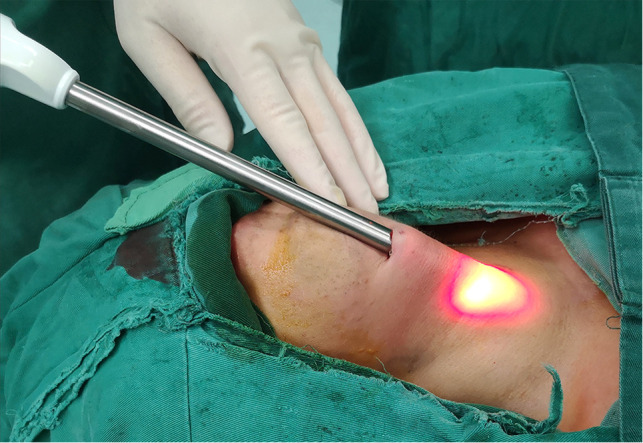
The visible subcutaneous stripper.

**Figure 3:**
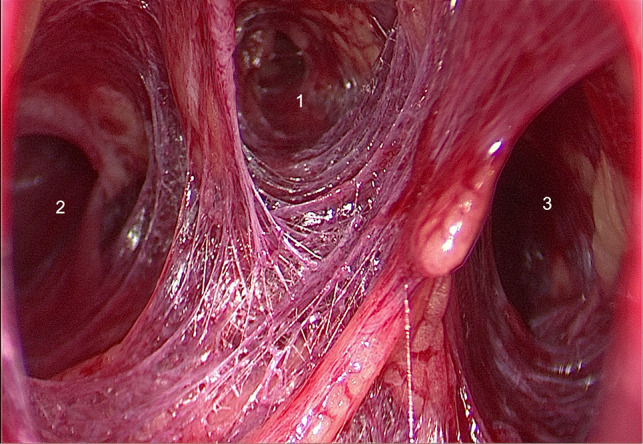
Three leading tunnels. 1: Tunnel in the middle of anterior cervical region; 2 and 3: Tunnels pointing to sternoclavicular joint.

**Figure 4:**
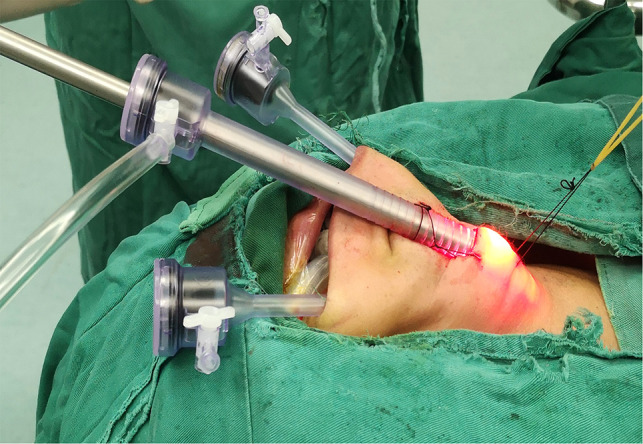
The distribution of three trocars: 10 mm troca was placed in the submental incision, two 5 mm trocas were placed laterally on the first molar.

(1) Following completion of the second sterilization of the oral and submental regions using iodophor, a 1.5-cm-long transverse incision was made two fingers below the mandible. Instead of a dilute epinephrine-saline solution (10) or normal saline (12), a visible subcutaneous stripper (Figure 2) was used to bluntly dissect and locate the three leading tunnels (Figure 3) via the first incision: one tunnel in the middle of the anterior cervical region, and the other tunnels pointing toward the sternoclavicular joints. All these operations were performed under the plane of the platysma muscle, which we named the first operative space and which is a key point for transoral thyroid surgery. (2) A 10-mm trocar was inserted into the submental incision for visualizing the surgical field, and CO_2_ was insufflated using this trocar to maintain a pressure of 4 mmHg and a flow rate of 30 L/min within the working space. Two 5-mm trocars were placed laterally on the first molar for manipulation (Figure 4). (3) Sharp dissection was then performed until the cervical white line was cut.

### Step Two

(1) Outside boundary: After the affected side of the thyroid gland was exposed, 0.1 mL of a carbon nanoparticle suspension was injected into the gland via percutaneous puncture to stain both the gland and the lymph nodes. Subsequently, sharp dissection was performed between the affected gland and the belt-shaped muscle to expose the carotid sheath and obtain the V1 signal. (2) Inside boundary: the prelaryngeal lymph nodes were removed, and the thyroid isthmus was divided from top to bottom.

### Step Three

**Figure 5:**
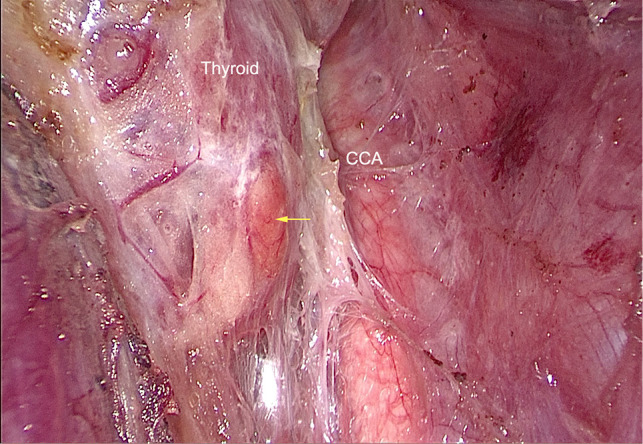
The Right superior parathyroid gland (The yellow arrow). CCA: Common carotid artery.

(1) The gland was separated upward from the cricothyroid space while cutting off a part of the belt-shaped muscle to ensure the complete dissection of the superior pole, looking carefully for the superior thyroid artery and the superior laryngeal nerve, and then closing off the superior thyroid artery. (2) Attempts were made to expose and keep the superior parathyroid gland in its original place (Figure 5).

### Step Four

This is another key point for transoral thyroid surgery. (1) Nerve probe forceps were used to locate the RLN within the larynx near Berry’s ligament, and when the R1 signal was obtained, the surrounding tissue was dissected bluntly to expose the RLN. (2) Berry’s ligament was divided, and the affected gland was then cut slightly and slowly while trying to protect the RLN and the inferior parathyroid gland, and it was packed into a sterile bag to remove from submental incision.

### Step Five

During this step, it is vital to assess whether the inferior parathyroid gland can be preserved at its original place. If not, it should be removed for autotransplantation (discussed in step seven).

### Step Six

**Figure 6:**
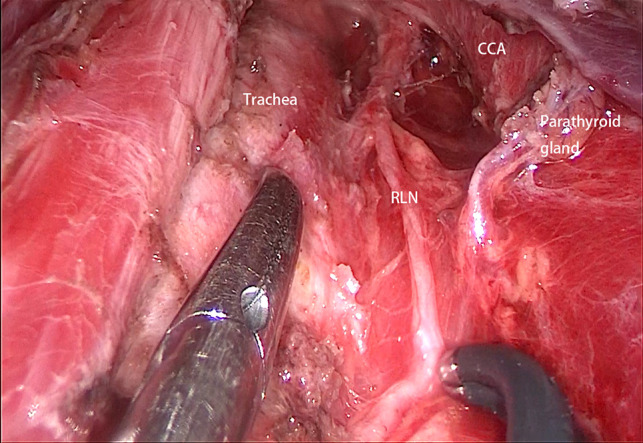
The central lymph node dissection was completed (right side). CCA: common carotid artery; RLN: recurrent laryngeal nerve.

For patients with PTC, ipsilateral central lymph node dissection was performed prophylactically. (1) Only the front of the left side of the RLN was exposed, while the right side was exposed totally. (2) The central lymph nodes were removed (Figure 6).

### Step Seven

(1) The area of the operation was washed with 1000 mL warm distilled water. (2) A 3-0 Quill absorbable suture was used to suture the belt-shaped muscle, and the drainage tube was placed in the submental incision. (3) All incisions were sutured with 4-0 absorbable sutures. (4) Autotransplantation: If it was deemed necessary to remove the parathyroid gland during step five, then during this step, the gland was injected into the left forearm of the patient.

### Postoperative Management

After completion of the surgery, patients were sent to the post-anesthesia care unit for further observation. Once the patients’ vital signs had been stabilized, they were sent back to the inpatient ward. A 3-day course of antibiotic prophylaxis (concentrated tinidazole gargles) was administered. The patients were started on a liquid diet 6 h after the operation, and the anterior cervical drainage tube was removed after 3 days. Outpatient follow-up was completed 1 month postoperatively.

SPSS Statistics 22.0 (IBM Inc., Armonk, New York, USA) was used to analyze the medical data of all patients enrolled in this study. Summary statistics (mean and standard deviation) are presented for continuous variables, while categorical variables are presented as absolute and relative frequencies (n, %). No formal study size calculation was performed. All cases who had undergone TOETSMVA at our hospital between November 2019 and to January 2020 were enrolled in the study.

## Results

The mean age of the 15 patients (8 women and 7 men) was 37 ± 10.8 years, and their mean body mass index was 22.3 ± 2.5 kg/m^2^ (Table 1). Preoperative ultrasonography revealed that the major tumors were distributed equally between the left (*n* = 8, 50%) and right side (*n* = 8, 50%), with one of the patients had bilateral tumors; the maximum and minimum diameters of the tumors were 1.9 and 0.5 cm, respectively, and there was no evidence indicative of lymph node metastases. The results of preoperative cytopathology revealed that eleven patients had PTC (VI) and four had suspicious PTC. The rate of *BRAF* gene mutations was 76.9%. No patient developed vocal cord paralysis before operation. All patients turned out to have PTC or papillary thyroid microcarcinoma upon postoperative pathology. Only one tumor (Case 10) had minimal extrathyroid extension, while the margins of all postoperative pathological specimens were negative. Seven patients (Cases 2, 3, 5, 6, 10, 14, 15) had central lymph node metastases (Table 1).

**Table 1:** Major perioperative examinations of those who underwent TETSMVA (*n* = 15).

Case	Age (years) /sex	BMI (kg/m2)	Preoperative ultrasound			FNA (Bethesda system)	BRAF mutation	Laryngofiber-oscopic examination	Postoperative pathology				
			Sizes (cm)	Major tumor location	LNM				Diagnosis	Maximun size(cm)	ETE	Margin	CLNM
1	54/F	22.7	1.4	Left;Middle	−	VI	+	Normal	PTMC	0.9	−	−	0/5
2	40/F	22.4	0.6	Right;Middle	−	V	+	Normal	PTMC	0.6	−	−	2/4
3	43/F	23.8	0.6	Left; /	−	VI	+	Normal	PTMC	0.5	−	−	1/8
4	26/F	19.3	0.9	Right;upper	−	V	+	Normal	PTMC	0.8	−	−	0/7
5	25/F	19.8	1.9	Right;lower	−	V	−	Normal	PTC	2	−	−	1/3
6	37/F	24.7	0.7	Right;upper Left;lower	−	VI	Left(−) Right(+)	Normal	Left:PTMC Right:PTMC	Left:0.3 Right:0.4	−	−	2/6
7	29/F	17.1	0.7	Right;lower	−	V	−	Normal	PTMC	0.7	−	−	0/8
8	56/M	25.6	0.8	Right;Middle	−	VI	/	Normal	PTC	1.1	−	−	0/1
9	29/M	24.2	0.6	Right;lower	−	VI	+	Normal	PTMC	0.3	−	−	0/3
10	34/M	25.1	0.9	Left;Middle	−	VI	+	Normal	PTMC	0.8	+ (Minimal)	−	4/8
11	43/M	19.1	0.5	Left;Middle	−	VI	+	Normal	PTMC	0.6	−	−	0/4
12	53/F	24.0	1.0	Left;Middle	−	VI	+	Normal	PTMC	0.7	−	−	0/5
13	31/M	21.4	0.5	Left;Middle	−	VI	+	Normal	PTMC	0.5	−	−	0/5
14	32/M	23.0	1.0	Left;Middle	−	VI	/	Normal	PTMC	0.9	−	−	8/11
15	24/M	21.8	1.1	Right;lower	−	VI	/	Normal	PTMC	0.8	−	−	2/3

*F, female; M, male; BMI, body mass index; LNM, lymph node metastasis; FNA, fine needle aspiration; PTC, papillary thyroid cancer; PTMC, papillary thyroid mircocarcinoma; ETE, extrathryoid extension; CLNM, central lymph node metastasis.*

The 15 surgeries (14 cases of thyroid lobectomy and a case of total thyroidectomy) were performed over 2 months. All patients underwent prophylactic central lymph node dissection on the affected side. The average duration of the surgeries was 146.5 ± 34.6 min and the mean intraoperative bleeding volume was 11.1 ± 6.3 mL. None of the surgeries converted to open thyroidectomy. The average volume of the anterior cervical drainage was 77 mL on postoperative Day 1. The mean duration of hospital stay was 3.3 ± 0.8 days. One patient developed transient RLN paralysis. No patient reported numbness of the skin in the center of the chin region or other postoperative complications (Table 2).

**Table 2:** The Introperative and postoperative details of those who underwent TETSMVA (*n* = 15).

Case	Date	Intraoperation					Postoperation					
		Extent of surgery	CLD	OT (min)	Blood loss	Convert to open thyroidectomy	Volume^a^ (mL)	Liquid diet(h)	Hospital day	Skin numbness^b^	Transient RLN paralysis	Other compli -cations
1	19/11/29	Lobectomy	+	150	20	−	105	6	3	−	−	−
2	19/12/4	Lobectomy	+	145	20	−	120	6	4	−	−	−
3	19/12/3	Lobectomy	+	107	10	−	75	6	3	−	−	−
4	19/12/10	Lobectomy	+	157	20	−	65	6	3	−	−	−
5	19/12/18	Lobectomy	+	128	5	−	65	6	3	−	−	−
6	19/12/19	Total	Left+ Right+	248	20	−	95	6	4	−	−	−
7	19/12/24	Lobectomy	+	150	10	−	75	6	3	−	+	−
8	19/12/24	Lobectomy	+	138	10	−	60	6	3	−	−	−
9	20/1/3	Lobectomy	+	144	10	−	75	6	4	−	−	−
10	20/1/14	Lobectomy	+	135	5	−	55	6	5	−	−	−
11	20/1/14	Lobectomy	+	170	10	−	130	6	3	−	−	−
12	20/1/15	Lobectomy	+	128	5	−	55	6	4	−	−	−
13	20/1/15	Lobectomy	+	122	5	−	40	6	2	−	−	−
14	20/1/21	Lobectomy	+	102	5	−	60	6	2	−	−	−
15	20/1/31	Lobectomy	+	174	10	−	80	6	3	−	−	−
Mean				146.5	11		77	6	3.3			

*CLD, centarl lymph node dissection; OT, operation time.*

*Volume^a^* *1st Day volume of the anterior cervical drainage, Skin numbness^b^* *sensory changes in the center of chin region.*

**Figure 7:**
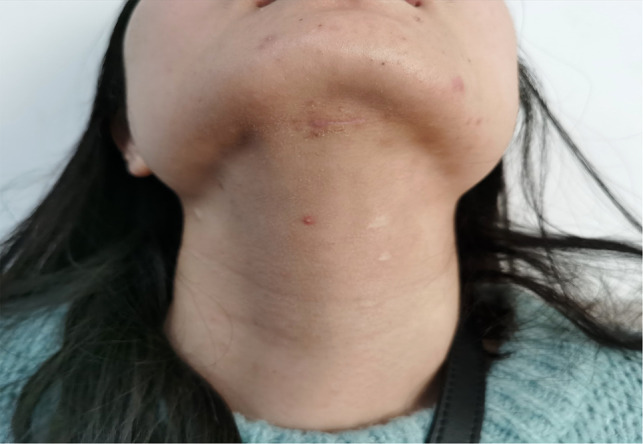
The postoperative pictures of a month.

During the follow-up, the submental incision healed well and was almost invisible one month after the operation (Figure 7). Since the incision was hidden under the chin, TOETSMVA had a satisfactory cosmetic effect. No signs of tumor implantation, recurrence, or metastases were apparent at the last follow-up.

## Discussion

Open thyroidectomy, developed by Emil Theodor Kocher in 1906, is the standard surgery for patients diagnosed with thyroid or parathyroid diseases (13). The anterior cervical scar caused by open thyroidectomy is distressing and stressful for patients, especially young women. Therefore, endoscopic thyroid surgery is a favorable alternative for some patients diagnosed with thyroid carcinoma.

In 1997, Hüscher et al. (14) performed the first endoscopic thyroid surgery. Thereafter, various approaches to performing thyroidectomy have been reported, such as the transaxillary approach (15), breast areola approach (16), and the bilateral axillo-breast approach (17). However, these approaches merely relocate the incision to other areas of the body, resulting in a more significant scar and more extensive tissue dissection. More importantly, blind spots have been previously reported during central lymph node dissection due to blockage by the sternum and clavicles in the breast approach (18).

In 2009, Wilhelm et al. (2) published the first series of transoral endoscopic thyroid surgery in eight patients. The surgery was performed via a sublingual approach combined with a vestibular approach, in which the observation port pierced through the floor of the mouth. There are several advantages of transoral thyroid surgery. First, the dissection route to the thyroid gland is short and direct compared with that in other approaches. Second, it is a truly scar-less method of thyroid surgery with three minimal incisions located in the mucosa of the oral vestibule through which all surgical procedures can be completed. Third, it offers a good surgical view and working space for manipulation, especially for the dissection of the lower central lymph nodes which are blocked by the sternum and the clavicles. Finally, when necessary, both thyroid glands can be completely removed without difficulty since the operative route lies on the midline.

However, it has some unavoidable limitations as well, for example, skin numbness in the center of the chin region and limited ability to extract large tumors. The mental nerve is the terminal branch of the inferior alveolar nerve, which lies between the anterior portion of the first premolar and the posterior portion of the second premolar. The rate of mental nerve injure was from 1% to 5% during TOETVA (19). Injury to a branch of the mental nerve during transoral thyroidectomy via the vestibular approach is inevitable since the 10-mm observation port is placed on the midline. Celik et al. (20) performed an anatomo-histological study on TOETVA, they found the median trocar went through a little numbers of small caliber branches of mental nerve. This means the injury of mental nerve by the median trocar is low but still possible. More importantly, the flap was separated from the observation incision to span the mandible resulting in postoperative edema, which is the main reason for skin numbness in related region. Zhang et al. (21) reported that TOETVA is associated with a higher jaw pain score (assessed using a visual analog scale) than open thyroidectomy. In our study, none of the patients developed skin numbness in the center of the chin region, neither were other reports (7, 8, 22), which indicates that TOETSMVA can circumvent the shortcomings of TOETVA.

All surgeries were completed successfully without conversion to open thyroidectomy. The mean operation time was 146.5 ± 34.6 min. The mean operation time of TOETVA with central lymph node dissection ranged from 180 to 207 min (11, 23, 24). According to our experience, there are two critical steps in the performance of transoral thyroid surgery for novice surgeons. The first one is the establishment of the first operative space, as described in step one of the procedure, and the second one is the dissection of Berry’s ligament, described in step four. The 10-mm observation port can be moved to the submental region in TOETSMVA, which reduces the difficulty in performing the required blunt dissection in the first operative space to some extent. The ability of TOETSMVA to provide a short and direct route to the thyroid gland that does not require stride over the jaw, constitutes an advantage that supports its use over other transoral endoscopic thyroid procedures. For novice surgeons who attempt to perform TOETSMVA, we suggest them choose patients with pointed chin, which might be benefit for the establishment of the first operative space.

There were two patients whose major tumor was located in the upper pole, which might be difficult for some surgeons to dissect the upper pole, based on our experience, we recommend that we can cut off a part of the belt-shaped muscle when we try to expose the upper pole.

## Conclusion

TOETSMVA is effective and safe in patients with thyroid cancer, and it does not result in skin numbness at the center of the chin region. This technique has advantages for the beginner of transoral endoscopic thyroid surgery, given its ability to provide a short and direct route to the thyroid gland which to some extent can reduce the difficulty of establishing the first operative space. However, our study was limited by the small sample size and short-term follow-up; thus, long-term oncological studies with a larger study sample are needed to verify our results.
